# Erdheim-Chester Disease with No Skeletal Bone Involvement and Massive Weight Loss

**DOI:** 10.1155/2017/3862052

**Published:** 2017-10-30

**Authors:** Hind Salama, Suleiman Kojan, Shaima Abdulrahman, Fahad Azzumeea, Ayman Alhejazi

**Affiliations:** ^1^Division of Hematology and HSCT, Department of Oncology, King Abdulaziz Medical City, Riyadh, Saudi Arabia; ^2^Department of Neurology, King Abdulaziz Medical City, Riyadh, Saudi Arabia; ^3^College of Medicine, King Saud bin Abdulaziz University for Health Science, Riyadh, Saudi Arabia; ^4^Department of Medical Imaging, King Abdulaziz Medical City, Riyadh, Saudi Arabia

## Abstract

Erdheim-Chester disease (ECD) is a rare type of non-Langerhans cell histiocytosis, with only 550 cases reported worldwide. ECD is characterized by diffuse histiocytic infiltration of multiorgans. The age of presentation of this disease is typically between 40 and 70 years. Bone disease is the most common symptom, as unique radiological findings of long bone sclerosis occur in 96% of cases. Furthermore, BRAF V600E mutation is detected in 60% of ECD cases. In this manuscript, we are describing a unique case of ECD; the patient is younger than most reported cases and has no bone pain or any skeletal involvement. This patient has unintentionally lost about 50% of his body mass and is suffering from progressive cerebellar manifestations with radiological evidence of cerebellar atrophy, in contrast to the usual ECD manifestation of cerebellar infiltration. In addition, the patient has cardiac, retroperitoneal, and perinephric involvement, but he retains his sexual drive and fertility. A tissue biopsy from the retroperitoneal mass displayed typical morphological and immunohistochemical features of ECD, and BRAF V600E mutation was detected. He was treated with pegylated interferon alpha, but his disease progressed and the treatment was changed to vemurafenib to which he had an excellent response at 6 weeks.

## 1. Introduction

ECD was first described in 1930 by William Chester and Jacob Erdheim. Since ECD is quite uncommon and is clinically diverse, diagnosis is frequently delayed and is possibly only confirmed at the autopsy. Diagnosis is usually based on radiological features and diffuse foamy histiocytic infiltrations of involved organs. The histiocytes are positive for CD68 and CD1a, but S100 is rarely positive. The presence of the BRAF V600E mutation further supports the diagnosis.

We are describing a unique case who presented with multisystem involvement mainly cerebellar, retroperitoneal, cardiac, and skin involvement, with no skeletal involvement and massive weight loss.

## 2. Case Presentation

We are reporting a case of a 34-year-old male who presented with a history of progressive unsteadiness, weakness, and slurred speech for the last three years. He reported bilateral arm weakness and difficulty in walking, getting out of chair, and standing up. He had rare episodes of choking. Although he claimed no loss of appetite, he lost 90 kilograms (kg) in the previous 3 years, and his baseline weight was 200 kg with a height of 180 cm. He also complained of polyuria and polydipsia but denied any visual impairment, headache, erectile dysfunction, palpitations, or orthostatic dizziness. He is working as a farmer. He is married and has one child and seven normal siblings.

A detailed examination revealed xanthelasmas around his right eye and anterior chest and abdominal wall. He has normal visual acuity and no Kayser-Fleischer ring or exophthalmos. Neurological examination showed normal cognitive function and normal cranial nerves. However, he presented with dysarthria, impaired coordination, and mild symmetrical weakness of the proximal arms and legs, and he needed assistance to get out of a chair. Laboratory investigations including a complete blood count as well as liver, renal, and thyroid function were within normal limits.

**Figure 1:**
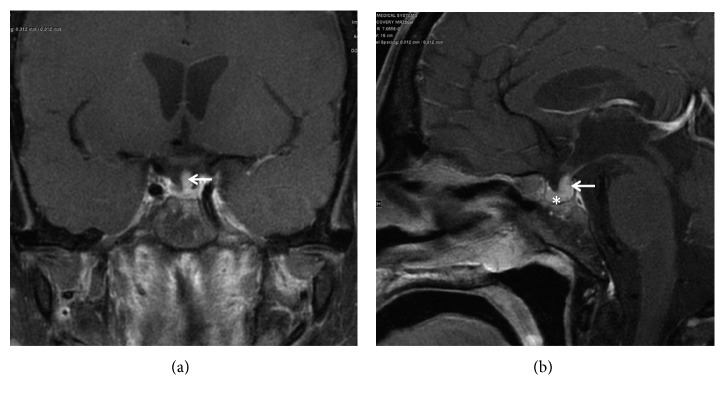
(a) Coronal and (b) sagittal pituitary MRI images post-gadolinium contrast show thickened enhancing pituitary stalk (arrow). It measures 4.7 mm at the level of the optic chiasm. The pituitary gland is within normal limits (asterisk).

Given the history of polyuria and polydipsia, diabetes insipidus (DI) was suspected. Diabetes insipidus is a shared feature of ECD and Langerhans cell histiocytosis, occurring in 25% of ECD patients. Further investigations to confirm cranial DI revealed serum osmolality of 324 mOsm/kg, serum sodium 140 mmol/L, urine osmolality 167 mOsm/kg, and Copeptin-proAVP(s) 2.4 Pmol/L, which are suggestive of central DI. The level of prolactin, LH, and FSH was normal. Pituitary magnetic resonance imaging (MRI) showed mild thickening of the pituitary stalk (Figure 1), diagnosis of cranial DI was confirmed, and he was started on desmopressin sublingually which dramatically improved his polyuria and polydipsia.

**Figure 2:**
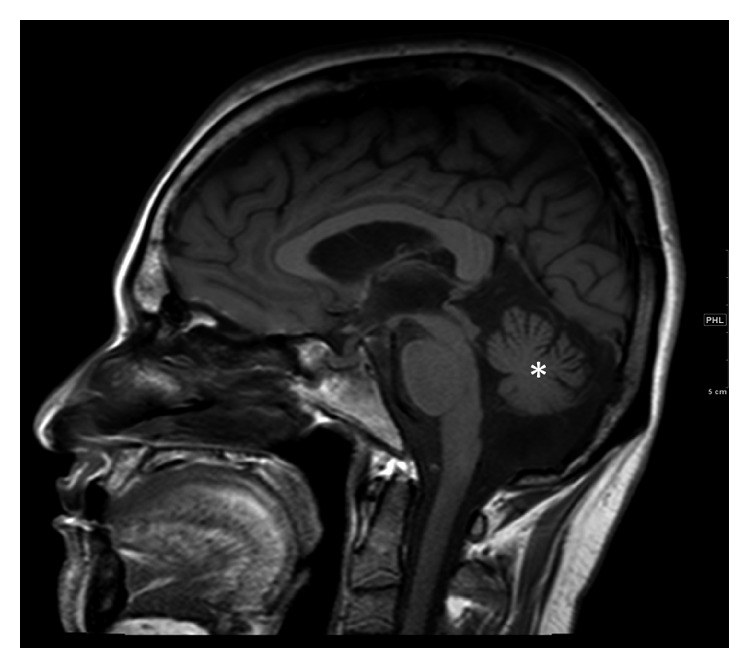
T1-weighted sagittal MRI of the brain shows uniform cerebellar loss.

A brain MRI revealed mild cerebellar volume loss and T2 hyperintensity predominantly in the right more than in the left cerebellar hemisphere and tegmental pons. There was symmetrical volume loss of the normally formed cerebellum associated with few bilateral confluent white matter T2/FLAIR hyperintensities involving the superior cerebellar and, to a lesser extent, the middle cerebellar peduncles, without diffusion restriction or enhancement (Figure 2). No infiltrative lesions were seen.

**Figure 3:**
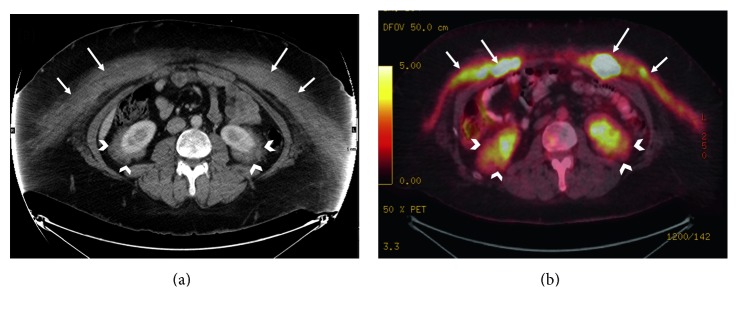
(a) Axial contrast-enhanced CT image of the abdomen and pelvis at the level of the kidneys shows bilateral rind-like perirenal soft tissue infiltrations, giving the appearance of “hairy kidney” (arrowheads). Anterior abdominal wall infiltration is also seen (arrows). (b) Corresponding PET/CT image shows intense FDG uptake in the same areas.

**Figure 4:**
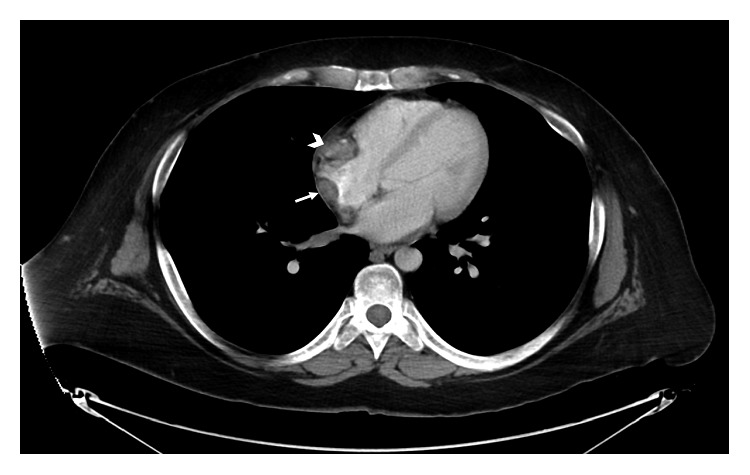
Axial contrast-enhanced CT image of the chest shows nodular soft tissue thickening overlying the right atrium posteriorly (arrow) and extending to the atrioventricular groove (arrowhead).

Computed tomography scan (CT) revealed bilateral perinephric soft tissue infiltration (hairy kidney sign), soft tissue thickening at the right atrioventricular sulcus, and infiltrative mass on the anterior abdominal wall (Figure 3(a)) as well as soft tissue thickening overlying the right atrium posteriorly (Figure 4). A cardiac MRI further confirmed a mass overlying the right atrium posteriorly which extended to the atrioventricular groove.

The orbit MRI was normal. A bone scan did not show abnormalities as well as X-rays of both femurs. The positron emission tomography (PET) (Figure 3(b)) showed intense hypermetabolic uptake in the muscles and skin of the anterior chest and abdominal wall and left axillary and inguinal lymph nodes as well as perinephric and cerebellar uptake.

**Figure 5:**
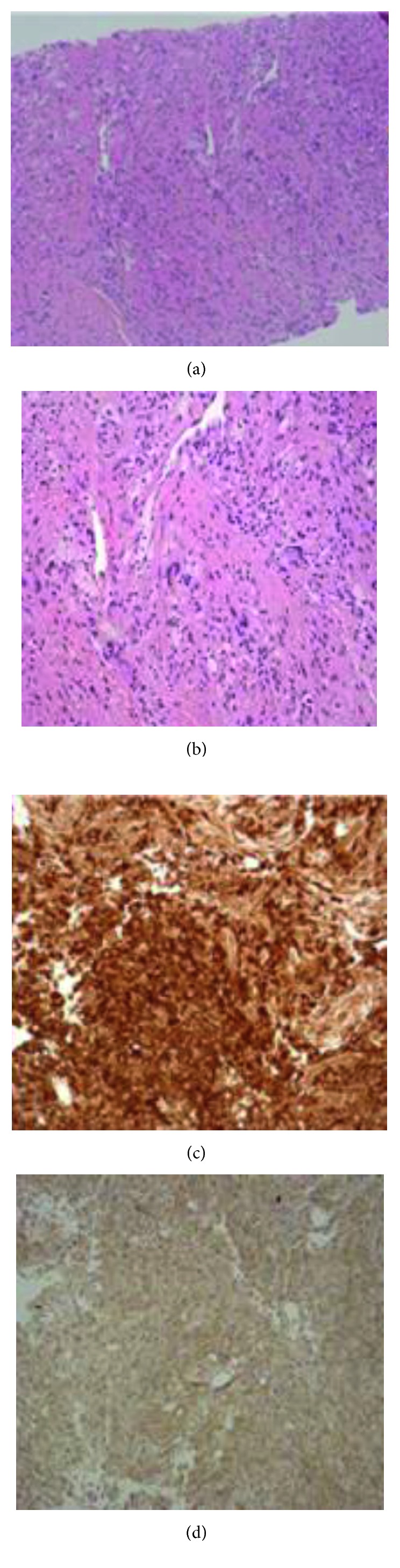
Histologically, the hematoxylin and eosin (H&E) stain shows infiltration by histiocytes (a), 100X. The infiltrate is composed of bland-looking histiocytes with foam-cell and giant-cell forms (b), 200X. The histiocytes stain positive for CD68 immunostain (c) and negative for S100 immunostain (d).

A biopsy from the anterior abdominal wall muscle showed infiltration with xanthomatous histiocytes and Touton multinucleated giant cells, positive for CD68 and negative for S100 and CD1a indicating xanthogranuloma (Figure 5). The tissue was sent to the bioreference laboratory (Gen Path) in the United States of America for sequencing of *BRAF* gene, and the BRAF V600E mutation was detected.

In conclusion, a young man with a three-year history of progressive weakness and unsteadiness was found to have cerebellar dysfunction, proximal muscle weakness, weight loss, and xanthelasmas as well as radiological and histological findings consistent with the diagnosis of ECD.

## 3. Discussion

ECD is an extremely rare type of non-Langerhans cell histiocytosis characterized by infiltration of multiple organs including the bones, central nervous system (CNS), heart, lungs, retroperitoneum, and skin. Skeletal involvement is the most frequent manifestation, with 96% of the patients having characteristic radiological findings of bilateral symmetric long bone sclerosis involving the diaphysis and metaphysis with sparing of the epiphysis [1]. Our patient had no skeletal involvement or bone pain.

CNS involvement is found in 30% of the patients [1], the most frequent sites involved are cerebellum and pons; however, cerebellar atrophy without infiltration as described in our case is also reported. Orbital infiltration and exophthalmos as well as pituitary infiltration and DI can also occur [2]. Our patient's MRI showed evidence of significant cerebellar atrophy. Laboratory investigations indicated features of DI.

Cardiac manifestations are also common but are often asymptomatic [10] and only discovered during routine imaging. The presence of cardiac involvement correlates with poor prognosis. The most frequent manifestation is the coated aorta which is visualized on CT as circumferential soft tissue sheathing of the thoracic and abdominal aorta. Other cardiac manifestations include pericardial effusions, pseudotumor of the right atrium in 30% of the cases, myocardial infarction, and pulmonary hypertension [3, 4].

Infiltration of perinephric tissue (“hairy kidney sign”), hydronephrosis, and retroperitoneal soft tissue lesions may occur. Liver and spleen involvement is rare, but if present, it correlates with a poor outcome [5]. Lung involvement occurs in 50% of patients in the form of pulmonary infiltration, pleural effusion, and a restrictive pattern when doing a pulmonary function test. Bronchoalveolar lavage usually contains macrophages and foamy histiocytes. Our patient had no CT findings, suggestive of lung involvement [6]. Skin xanthelasmas, mainly as eyelid plaques, can also involve the face, neck, axilla, trunk, and groin. Histologically, there is proliferation of foamy lipid-laden histiocytes and surrounding Touton giant cells. The results of immunohistochemical (IHC) staining for CD68, CD163, and FACTOR XIII are positive, with CD1a and Langerin testing being negative. S100 is rarely weakly positive. ECD histiocytes are morphologically and immunohistochemically identical to those in juvenile xanthogranuloma (JXG). ECD is considered as a variant of JXG. The BRAF V600E mutation is present in more than 50% of cases, with other mutations such as *N/KRAS* and *PIK3CA* described in *BRAF*-negative ECD [7].

Our patient had CNS, cardiac, skin, and retroperitoneal involvement without skeletal, orbital, or pulmonary involvement. The tissue biopsy supported ECD, and the BRAF V600E mutation was detected.

He developed his symptoms shortly after his marriage; however, he maintained his libido, erectile function, and fertility. He has a one-year-old healthy son. There is limited literature about fertility and ECD, although erectile dysfunction and gonadotropin insufficiency are possible complications [5, 8]. Most ECD patients remain undiagnosed for many years due to the rarity of the disease and diverse presentation. Our patient was diagnosed less than a year from his presentation to the subspecialty clinic.

Various treatment options have been used in ECD with limited success. Interferon alpha and pegylated interferon alpha are currently the therapeutic modalities supported by many clinical trials, with higher doses needed for those with CNS and cardiac involvement [5, 9]. Anticytokine-directed therapies such as anakinra and infliximab as well as corticosteroids and imatinib have been tried with a lower success rate than interferon [5]. Vemurafenib, an inhibitor of BRAF V600E, has shown an excellent response in *BRAF*-mutated ECD refractory to interferon. It resulted in radiological and clinical improvement and should be considered as a second line of treatment for patients with detected *BRAF* mutation [4, 5].

Based on a study performed on 80 patients with ECD in France, those who are *NRAS* or *PIK3CA* mutated present with multiorgan involvement compared to patients with mutated *BRAF* or the wild-type *BRAF/NRAS/PIK3CA*, although a larger number of patients are required to confirm this result [7].

ECD patients with *RAS* mutations might benefit from anti-MEK therapy such as binimetinib or trametinib. Combinations of anti-BRAF and anti-MEK have benefited patients with advanced melanoma who are *RAS* mutant [7, 11]. Future studies targeting this pathway in ECD are needed.

**Figure 6:**
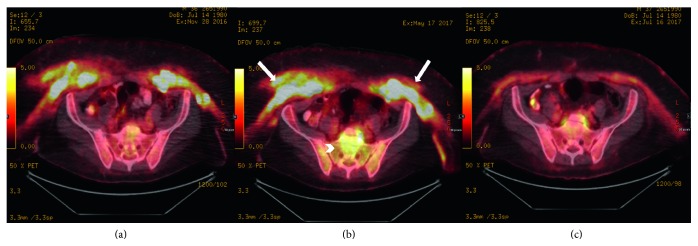
Series of FDG-PET/CT images of the abdomen and pelvis at the level of the sacrum throughout the management course. (a) Initial PET scan at the time of presentation (November 2016). (b) Progression of anterior abdominal wall involvement (arrows) and new sacral involvement (arrowhead) while on pegylated interferon (May 2017). (c) Significant interval regression of sacral and anterior abdominal wall involvement after starting vemurafenib (July 2017).

**Figure 7:**
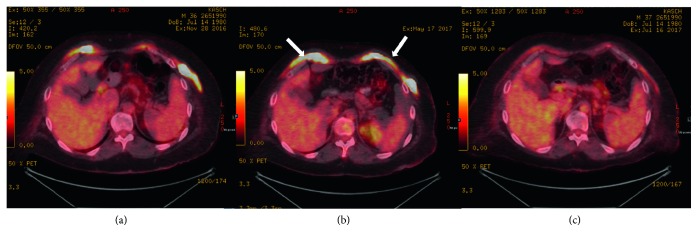
Series of FDG-PET/CT images of the chest at the level of T11 throughout the same period. (a) Initial PET scan at the time of presentation (November 2016). (b) Progression of lower anterior chest wall involvement (arrows) (May 2017). (c) Interval regression of lower anterior chest wall involvement after starting vemurafenib (July 2017).

Cladribine has been studied and found effective in refractory patients who lack the *BRAF* mutation [6]. Our patient was started on pegylated interferon alpha at a dose of 180 micrograms weekly. Unfortunately, after three months, his cardiac mass remained the same on cardiac MRI while repeated PET scan showed interval increase in metabolic activity of the anterior abdominal and chest wall muscles (Figure 6), as well as development of new axillary lymphadenopathy. A brain MRI showed significant cerebellar atrophy. He was then started on vemurafenib 480 mg twice per day. After six weeks of therapy, he showed clinical and radiological improvement. A repeated PET scan after six weeks of vemurafenib (Figures 6 and 7) showed significant interval regression in the cutaneous lesions and perinephric lesions as well as the disappearance of the axillary lymphadenopathy and slight regression in the right atrial soft tissue thickening. Our plan is to continue on vemurafenib until disease progression.

## 4. Conclusion

ECD is an extremely rare disease, with heterogeneous and diverse clinical manifestations. Atypical presentation can be challenging.

This case is unique for many reasons: the patient had severe constitutional symptoms manifested as weight loss of 90 kg, no skeletal involvement, and cerebellar atrophy without clear infiltrations at presentation. He preserved his fertility and libido, and lastly, he is younger than currently reported cases with typical age presentation of 40–70 years.

Without treatment, ECD is fatal. The estimated survival is 3 years. Even with therapy, the outcome is poor especially in patients with CNS manifestations. Clinical trials are needed to explore improved therapeutic options.

## References

[1] Mazor R. D., Manevich-Mazor M., Shoenfeld Y. (2013). Erdheim-Chester disease: a comprehensive review of the literature. *Orphanet Journal of Rare Diseases*.

[2] Wright R. A., Hermann R. C., Parisi J. E. (1999). Neurological manifestations of Erdheim-Chester disease. *Journal of Neurology, Neurosurgery, and Psychiatry*.

[3] Ponsiglione A., Puglia M., Barbuto L. (2015). Cardiac involvement in Erdheim-Chester disease: MRI findings and literature revision. *Acta Radiologica Open*.

[4] Haroche J., Cohen-Aubart F., Emile J.-F. (2013). Dramatic efficacy of vemurafenib in both multisystemic and refractory Erdheim-Chester disease and Langerhans cell histiocytosis harboring the BRAF V600E mutation. *Blood*.

[5] Diamond E. L., Dagna L., Hyman D. M. (2014). Consensus guidelines for the diagnosis and clinical management of Erdheim-Chester disease. *Blood*.

[6] Azadeh N., Tazelaar H. D., Gotway M. B., Mookadam F., Fonseca R. (2016). Erdheim Chester disease treated successfully with cladribine. *Respiratory Medicine Case Reports*.

[7] Emile J.-F., Diamond E. L., Hélias-Rodzewicz Z. (2014). Recurrent RAS and PIK3CA mutations in Erdheim-Chester disease. *Blood*.

[8] Tritos N., Weinrib S., Kaye T. (1998). Endocrine manifestations of Erdheim–Chester disease (a distinct form of histiocytosis). *Journal of Internal Medicine*.

[9] Braiteh F., Boxrud C., Esmaeli B., Kurzrock R. (2005). Successful treatment of Erdheim-Chester disease, a non–Langerhans-cell histiocytosis, with interferon-α. *Blood*.

[10] Nicolazzi M. A., Carnicelli A., Fuorlo M., Favuzzi A. M., Landolfi R. (2015). Cardiovascular involvement in Erdheim–Chester disease: a case report and review of the literature. *Medicine*.

[11] Eroglu Z., Ribas A. (2016). Combination therapy with BRAF and MEK inhibitors for melanoma: latest evidence and place in therapy. *Therapeutic Advances in Medical Oncology*.

